# The impact of national guidelines on venom immunotherapy practice in the United Kingdom

**DOI:** 10.1111/cea.12728

**Published:** 2016-04-26

**Authors:** L. Diwakar, P. Ewan, P. A. J. Huber, A. Clark, S. Nasser, M. T. Krishna

**Affiliations:** ^1^ University of Birmingham and University Hospitals BirminghamBirminghamUK; ^2^ Allergy clinicAddenbrookes HospitalCambridgeUK; ^3^ BSACILondonUK; ^4^ Heart of England NHS Foundation Trust and Aston UniversityBirminghamUK

Venom immunotherapy (VIT) is the only effective treatment for prevention of further anaphylactic reactions to bee and wasp stings in allergic individuals. A previous national survey carried out in 2006/2007 revealed significant heterogeneity in UK practice 1. The British Society for Allergy and Clinical Immunology (BSACI) guidelines for the diagnosis and management of hymenoptera venom (HV) allergy were subsequently published in 2011 2 (Table 1).

**Table 1:** Results of the VIT UK national surveys

	2006/2007^b^ (%)	2014 Survey (%)			
	Overall *n* = 48	Overall *n* = 64	Allergists *n* = 28	Immunologists *n* = 21	Other *n* = 15
Diagnosis of HV allergy					
SPT is the first‐line test (%)	45	66	77	65	46
SPT highest concentration is 100 mcg/mL (%)	43	36	46	15	54
SPT highest concentration is 300 mcg/mL (%)	55	62	54	85	36
If sSIgE^a^and SPT negative, IDT will be performed by (%)	50	80	91	85	54
IDT highest concentration is 1.0 mcg/mL (%)	45	81	74	94	71
Component‐resolved tests performed if dual positive for wasp and bee venom sSIgE and skin tests (%)	NA^c^	87	81	95	77^d^
Baseline tryptase (bT) checked in all patients with a history of systemic reaction (%)	47	88	85	100	70
Administration of VIT					
Conventional up‐dosing protocol preferred (%)	92	74	55	90	82
Ultra‐rush/rush induction protocol ever used (%)	25	38	60	21	27
Antihistamines for all VIT patients before injection (%)	42	40	53	22	46
Maximum interval between injections during maintenance is 4–8 weeks (%)	89	94	90	94	100
Target maintenance dose is 100 mcg/mL (%)	89	98	100	94	100
Patients monitored for at least 1 h post‐injection (%)	NA^c^	88	90	94	73
Optimal duration of VIT is 3 years (%)	55	83	84	94	64
VIT extended if sSIgE^a^ detected at the end of 3–5 years (%)^e^	14	6	5	5	27

a Serum‐specific IgE.

b Results from the 2006/2007 survey.

c This question did not feature in the previous survey.

d An additional 9% would refer patient to tertiary centre.

e Not recommended in the BSACI guideline.

This repeat survey was carried out to assess whether publication of BSACI guidelines helped improve the diagnosis and management of HV allergy in the UK National Health Service (NHS).

## Methods

The survey questionnaire (same as in previous survey 1 with two additional questions, Appendix 1) was emailed *via* a web link (www.surveymonkey.com) to all consultant physicians who were members of the BSACI in March 2014. A total of 247 practitioners from 80 UK NHS hospitals were contacted. Respondents were requested to identify their speciality interest as well as whether they were adult or paediatric physicians.

Two reminder emails were sent in April/May 2014.

The results were compared with the data obtained during the 2006/2007 survey to assess the impact of the BSACI guideline on the practice of HV allergy in UK NHS allergy services.

## Results

A total of 113 (46%) responses were received. One hundred and seven (95%) respondents worked in 75 centres across the United Kingdom. Two respondents were from the republic of Ireland and three UK respondents did not divulge their centre of work. One responder was not practising in the United Kingdom at the time of the survey and was therefore excluded from analysis.

Over half of the respondents of this resurvey (*n* = 67/113; 59.3%) carried out VIT. Of these, 64 responses received from 35 UK centres were deemed eligible for analysis.

Amongst the 64 respondents undertaking VIT, 28 (44%) were adult or paediatric allergists, 21 (33%) were immunologists and 15 (23%) identified themselves as ‘other’ clinicians. A majority of the latter group (*n* = 10) were respiratory physicians with an interest in allergy, whilst three were paediatricians with an interest in allergy.

### Clinic structure

A total of 37 (58%) respondents managed only adults with allergy, 12 (19%) only treated children, whilst 15 respondents (23%) managed both adults and children at their clinic.

**Figure 1:**
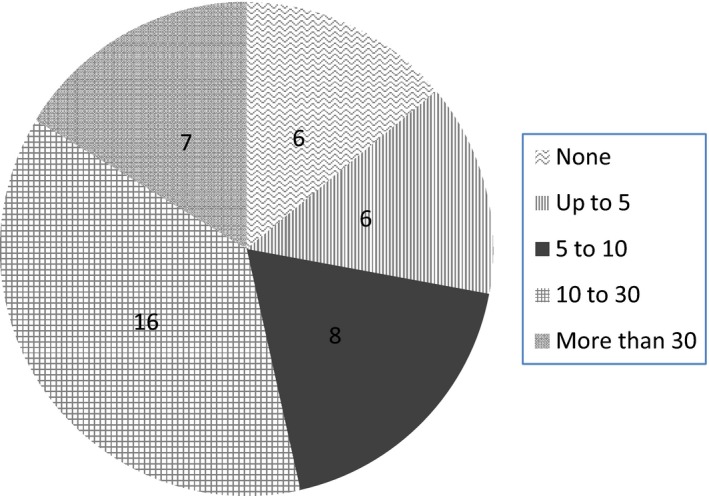
Number of centres as per VIT patient load.

Six centres had no patients undergoing VIT, whilst seven had more than 30 patients undergoing VIT at the time of the survey (see Fig. 1). Just over half the respondents (52%) had 10 or more patients undergoing VIT at their centre at the time of the survey. Most clinics were staffed by a specialist nurse (86%) and/or by a consultant (76%). Some centres (44%) had junior doctors in the clinic.

### Diagnosis of HV allergy

The table provided summarizes the clinical practice relating to the diagnosis of HV allergy. About two‐thirds of the respondents use skin prick tests (SPTs) as the first‐line investigation for diagnosis and a majority carried out intradermal tests (IDTs; 80%) and/or component‐resolved diagnostic (CRD) tests (85%) when skin prick and serum‐specific IgE tests do not provide a clear diagnosis. Baseline serum tryptase (bT) measurement is also requested by most respondents (88%) in all patients presenting with a systemic reaction to HV, irrespective of severity.

### Safety of VIT practice

Almost all respondents check the patients' identity at each visit and ensure that changes to medication are regularly documented. Only about 65% of respondents check pulse and blood pressure before every injection. 90% check peak expiratory flow rate (PEFR) prior to VIT injections. Most (88%) monitor patients for a minimum of an hour post‐VIT injection.

Most physicians (92%) consider individuals with severe, uncontrolled or brittle asthma unsuitable for VIT. Ongoing beta‐blocker and ACE inhibitor therapy is considered as a contraindication by most (78% and 62%, respectively), whereas most physicians (88%) are happy to initiate VIT in patients with an elevated bT.

The data pertaining to administration of VIT are summarized in the table. The majority prefer conventional up‐dosing protocol (76%) aiming for a maintenance dose of 100 mcg (98%) and administer maintenance injections at a 4‐ to 8‐weekly interval (94%).

### VIT protocols

Conventional protocol (12‐week gradual up‐dosing) is favoured by most (76%), although rush (4–7 days) and ultra‐rush (1–2 days) up‐dosing are preferred by 22%. Forty percent of respondents routinely use pre‐medication with antihistamines in all patients undergoing VIT, whereas the others only used antihistamines in patients experiencing allergic reactions to VIT.

Ninety percent offer VIT for 3 years, whereas the others routinely continue treatment for 5 years.

A third and none of the respondents check serum‐specific IgE and serum‐specific IgG4 antibodies to HV, respectively, at the end of the VIT programme. Five percent of the respondents extended VIT on the basis of a positive serum‐specific IgE test to insect venom at the end of 3 years.

### VIT survey results from 2006 to 2007 1

In the survey carried out in 2006/2007, 53 responses were received in response to 86 online questionnaires (61.6% response rate). Serum‐specific IgE was more frequently used as a first‐line investigation (55%) compared to SPTs (45%). Only half the respondents carried out intradermal tests when initial tests were negative. Fifty‐three percent checked bT in individuals with systemic reactions to insect venom. Ninety‐two percent preferred conventional up‐dosing protocols for VIT and 56% carried out VIT for 3 years.

Since the last survey, there has been a statistically significant improvement in the use of skin tests as first line of diagnosis (*P* = 0.05, chi‐square test, 1 df), in the use of intradermal tests when skin and specific IgE tests are not conclusive (*P* = 0.004, chi‐square test, 1 df) and in testing baseline tryptase levels when an individual presents with a systemic reaction to insect venom (*P* < 0.00001, chi‐square test, 1 df).

## Discussion

The current survey was sent to practitioners across 80 UK NHS trusts. Responses were received from 72 trusts. Thus, the data obtained are a credible snapshot of current UK NHS practice.

The response rate was moderately good in this survey. However, response rate was marginally higher in the 2006/7 survey (62%), although the absolute number of responses was lower (53 responses). Of the respondents, 48 (85%) carried out VIT at their centre 1 as opposed to 59.3% in the current survey. A few questions were added to the 2006/2007 questionnaire to reflect developments in the practice of VIT and also to improve the breadth of data collection. For example, data regarding speciality of the respondents or data related to the use of CRD tests were not collected in the previous survey.

Although the results of the current survey are not directly comparable with the results from the 2006/2007 study 1, the diagnostic practices for HV allergy in the United Kingdom appear to have improved following publication of the BSACI guidelines 2 (see Box 1).

Box 1 **Examples of improved practice since last survey (In keeping with BSACI guidelines)**

- There has been a significant increase in the number of practitioners who carried out SPTs (66% vs. 45% in 2006/2007), IDTs (80% vs. 50% in 2006/2007) and check bT (88% vs. 47% in 2006/2007) in keeping with the BSACI guidelines.
- A majority of practitioners offer VIT to patients with an elevated bT.

Component‐resolved diagnosis has become an invaluable tool enhancing the accuracy of diagnosis, particularly in patients with dual sensitization to bee and wasp venoms 3. It is encouraging to note that a majority (87%) of the respondents use these tests in diagnosis of HV allergy as per the guideline recommendations.

Our data have also highlighted significant deviations from recommendations made in the BSACI guideline regarding diagnosis of HV allergy (see Box 2).

Box 2 **Deviations from BSACI guidelines**

- One‐third do not use SPTs as the primary diagnostic tool, and about 20% do not carry out IDT at their centre when SPT and serum‐specific IgE are negative.
- 62% of the respondents use concentrations up to 300 mcg/mL for SPTs and 15% use up to 10 mcg/mL for IDTs, which are higher than those recommended in the BSACI guideline
- 12% of respondents do not check bT in all patients with a systemic reaction to HV.
- 12% do not monitor patients for an hour after administration of VIT injection.
- 35% do not measure pulse and blood pressure prior to the VIT injection.
- 60% of the respondents do not offer VIT to individuals on ACE inhibitor therapy.
- 5% may continue VIT beyond 3 years based on a positive serum‐specific IgE result.

Concomitant use of beta‐blockers can potentially interfere with the therapeutic efficacy of adrenaline and cause refractory or protracted anaphylaxis. The BSACI guideline therefore recommends that these drugs are substituted or temporarily withdrawn following a risk–benefit analysis in the individual patient 2. A majority of the respondents were unwilling to offer these patients VIT at their centre. Use of ACE inhibitors, on the other hand, is not associated with an increased risk during VIT, but is a recognized risk factor for anaphylaxis to field stings 2. Nevertheless, it is interesting to note that over 60% of the respondents would not initiate VIT in these patients. These data suggest that individuals at high risk of anaphylaxis are being denied an effective, long‐term treatment for their venom allergy. A recent position paper from the European Academy of Allergy and Clinical Immunology (EAACI) concluded that concomitant treatment with a beta‐blocker is not a contraindication for VIT, whereas ACEi should be substituted with an alternative agent where feasible^3^. These points should be revisited in the next iteration of the BSACI guideline.

Although some aspects of VIT have improved in the United Kingdom, others remain unchanged since our last survey; a few of these (e.g. not checking bT in patients with systemic reactions, inadequate monitoring following VIT injections) could potentially compromise patient safety.

Several factors may affect uptake of a guideline. These include quality of evidence underlying the guidance, extent of promotion and awareness of the guidance and commitment of individual trusts to guideline implementation 4. Studies in other areas have suggested that centres with high patient throughput show better adherence to guidelines 5, although no such differences were found in our survey. Reduced uptake in some aspects (such as the use of 100 mcg/mL vs. 300 mcg/mL solutions for SPT, measurement of PEFR and blood pressure prior to administration of VIT injection) can be explained in part by conflicting expert opinions. However, these explanations cannot be applied to all other aspects of the guidance for which the uptake was less than satisfactory. Previous explorations regarding effectiveness of guidelines have not been able to suggest that specific interventions improve uptake 6, 7. However, it has been suggested that regular dissemination of educational material and discussion in short (e.g. lunchtime) meetings 6, active clinician engagement using interactive educational sessions and automated clinical reminders 7 may all be practical and useful in guideline implementation. Improved dissemination can improve equity and provision of high standards of care, which are the stated objectives of NHS England Specialised Services.

## Strengths and limitations

This is the first report assessing the impact of a national guideline on allergy services in the published literature. The British Society for Allergy and Clinical Immunology is the largest professional body for allergists and clinical immunologists in the United Kingdom. Venom immunotherapy in the United Kingdom is administered only in secondary care under specialist supervision. The initial survey was carried out prior to publication of the BSACI guidelines in 2011. However, there have since been two other significant publications that could have affected UK VIT practice. These include the NIHR HTA guidance for the use of Pharmalgen in VIT 8 as well as a systematic review from the Cochrane collaboration highlighting the effectiveness of VIT in the prevention of allergic reactions to insect stings 9.

The response rate for the current survey was lower (46%) although the number of respondents was greater than in the previous survey. Only 59% of those who responded (27% overall) practised VIT during the recent survey. It is worth stating that this is comparable to the response rate of the previous survey (56%) and that of other national UK allergy surveys 10.

In addition, there was no classification of practitioners into the allergy, immunology or other specialist groups. The results are presented based on the self‐identification of respondents into these categories in the recent survey questionnaire.

The previous survey was sent only to 81 UK practitioners identified as practising VIT from the BSACI website. The membership has since grown and database updated and therefore the current cohort (247 members) is not directly comparable to those surveyed in 2006/2007. Respondents chose not to answer some questions in the survey. Where data were missing, this was ignored. This approach may have potentially introduced some bias into the interpretation of the data.

As with any survey, these data are subject to response bias (i.e. the tendency of respondents to provide responses considered ‘acceptable’ rather than those that reflect their true practice). However, it is hoped that this was minimal given that in our survey, the respondents were made aware of the confidential nature of the responses upfront.

The survey may also be subject to a selection bias, as only those individuals with some familiarity with the guidelines may have responded to the survey.

## Unanswered questions and future research recommendations

The data from this survey have been used to assess the uptake of VIT guidelines in the United Kingdom. Respondents may be directly asked for their opinion regarding existing guidelines in future surveys. This would help improve the quality and uptake of future guidelines.

To understand the effect of centre size on practice, future surveys could be made centre‐focussed rather than focussing on individual practice where appropriate. Also, it may be useful to have generic sections in the questionnaire as well as separate sections aimed at adult and paediatric practices since practice of allergy in children can be different from that of adults in some areas (e.g. use of intradermal tests, checking blood pressure before injections, issues around the use of beta‐blockers and ACEi).

To assess patient selection criteria in accordance with the NIHR HTA recommendations 8, future surveys should include questions about the use of generic 11 or specific 12 quality of life (QoL) questionnaires before commencement and after completion of VIT.

## Conclusion

Despite above‐stated limitations, the current survey suggests better adherence to best practice parameters in several domains of diagnosis and management of HV allergy. However, the data also highlighted the following areas where further improvement is needed: use of SPTs as the first‐line investigation, use of optimal allergen concentrations for skin tests, checking bT in all patients with systemic reactions to HV, and monitoring patients for an optimal duration following VIT injections. Updating guidelines regularly and having a plan for dissemination may improve adherence.
